# Physician-Level Determinants of Cervical Cancer Screening Practices: A Socio-Ecological Model-Based Study from Adjara, Georgia

**DOI:** 10.3390/healthcare14070961

**Published:** 2026-04-06

**Authors:** Koba Kamashidze, Tina Beruchashvili, Tamar Peshkova, Irina Nakashidze, Liana Jashi, Sarfraz Ahmad

**Affiliations:** 1 School of Health Sciences, University of Georgia, 0171 Tbilisi, Georgia; kobba77@gmail.com (K.K.); t.beruchashvili@ug.edu.ge (T.B.); 2 Faculty of Natural Sciences and Health Care, Batumi Shota Rustaveli State University, Ninoshvili St. No. 35, 6010 Batumi, Georgia; tamar.peshkova@bsu.edu.ge; 3 Department of Clinical Medicine, Avicenna-Batumi Medical University, 6010 Batumi, Georgia; liajashi@yahoo.com; 4 Gynecologic Oncology Program, AdventHealth Cancer Institute, 2501 N. Orange Ave., Suite 786, Orlando, FL 32804, USA

**Keywords:** cervical cancer, diagnosis, screening services, prevention, treatment options, physician-led factors, cost-effectiveness, socio-ecological factors, data-survey, practice pattern, autonomous Republic of Adjara, Georgia

## Abstract

**Background/Objectives:** Cervical cancer is widely recognized as a preventable disease; however, participation in screening programs remains insufficient in many transitional health systems. In the Georgia, organized screening services are available, yet utilisation remains low, indicating barriers to screening extend beyond access alone. This study, therefore, examined physician-level factors influencing the promotion of cervical cancer screening in the Adjara region of Georgia, with focus on routine clinical practice and organizational conditions. **Methods:** A cross-sectional survey was carried out among physicians providing outpatient and preventive services in six municipalities of the Adjara region. The analysis was guided by a socio-ecological framework and examined individual, inter-personal, and organizational factors in relation to physicians’ recent cervical cancer screening recommendation practices. Multivariable logistic and ordinal regression analyses were used to identify factors associated with screening promotion. **Results:** Despite a generally high level of support for cervical cancer screening among physicians, regular screening recommendations were not consistently reported. Limited consultation time, uncertainty regarding screening-related harms, and rural practice settings were independently associated with a lower probability of having recently recommended screening. In contrast, favourable attitudes toward screening on their own were not sufficient to translate into routine preventive practice. **Conclusions:** These findings indicate that gaps between physician attitudes and screening promotion are largely driven by structural and organizational factors rather than a lack of professional support. Efforts to reduce workflow constraints, improve clarity around screening guidance, and integrate preventive counselling into routine clinical practice may be essential for improving screening uptake in similar healthcare system contexts.

## 1. Introduction

Despite many advances, cervical cancer remains a major global health issue due to high incidence and mortality [1,2], as it causes approximately 350,000 deaths each year [3,4]. Cervical cancer is a heterogeneous and complex disease [5,6] which is caused by genetic, epigenetic [7], and multiple microenvironmental factors [8], which often complicates the process of diagnosis, prognosis, and treatment [9,10]. Despite its complexity, cervical cancer is preventable [3,11]; however, the low- and middle-income countries bear a disproportionate burden due to gaps in vaccination, screening, and treatment options available [12,13]. Therefore, addressing these disparities with effective policies and resources is crucial in reducing the incidence and mortality globally [14].

Screening of individuals with cervical cancer should include human papillomavirus (HPV) testing. In the case of a positive HPV status, further genotyping and cytological evaluations are necessary to assess the risk of cervical precancer fully. It is worth noting that the HPV testing during adolescence significantly reduces the incidence of cervical pre-cancer or cancer (in many cases by up to 90%) [4]. Vaccination serves as the primary method for the prevention of cervical cancer, and Eastern Europe/post-Soviet health systems represent a specific gap in evidence, whereas cervical cancer screening measures are necessary for its secondary prevention [4,15].

The complexity of the disease and its causative factors make the screening difficult. Timely identification of individuals with high-risk HPV genotypes increases the likelihood of prevention and early detection, which remains an essential means for reducing mortality rates. The availability of cervical cancer prevention and screening devices/services are barriers related to the administration process [16,17]. The specifics of these barriers may vary across countries, creating unique challenges for delivering adequate, comprehensive prevention and screening services [3,18]. Depending on the capacity of the existing healthcare infrastructure, access to screening clinics, confirmed diagnosis, and appropriate treatment may be associated with a number of barriers [19]. According to Pimple et al. [14], it is crucial to develop uniform and cost-effective strategies to reduce the burden of cervical cancer worldwide, which may help reduce the existing inequalities in the burden of cervical cancer between low- and high-income/resource countries [19].

However, prevention is also achievable with effective strategies for early detection. The World Health Organization (WHO) is committed to eliminating HPV vaccination, supporting the implementation of appropriate screening, etc. However, to achieve the WHO goals, it is necessary to have a framework for monitoring the implementation of screening programs [3]. Even though the current approach to cervical cancer prevention and screening has been developed, it should be linked to the large-scale implementation of a population-based cervical cancer screening/prevention program [20]. Despite many achievements, cervical cancer screening practices are critically important for early detection of the disease and the guidance of appropriate treatment [21], providing opportunities to significantly reduce both cervical cancer incidence and mortality [22].

Despite having a well-developed primary healthcare network, the autonomous Republic of Adjara (in Georgia), consistently experiences low participation in national screening initiatives of the country. Despite the availability of effective screening methods, many barriers hinder their use, including a lack of knowledge and awareness, inadequate training, and unclear understanding of the program procedures, and accessibility restrictions, etc. [23,24]. Therefore, this study attempts to address the complex barriers that require significant adaptation and tailoring of approaches to the needs of specific populations, thereby significantly improving screening rates and reducing the national/global burden of cervical cancer.

## 2. Materials and Methods

### 2.1. Study Population

A cross-sectional analytical survey of physicians (n = 209) was conducted between October and December 2024 in the Autonomous Republic of Adjara, Georgia, specifically in the Southwestern region, which comprises six municipalities (Batumi, Keda, Khelvachauri, Khulo, Kobuleti, and Shuakhevi). A structured questionnaire, adapted from validated instruments and pilot-tested, was designed to assess factors at the individual, interpersonal, and organizational levels. It should also be noted that the primary outcome was self-reported cervical cancer screening within the past week, while the secondary outcome was the frequency of screening recommendations. Multivariable logistic and ordinal regression analyses were used to examine factors associated with screening recommendation practices.

The study forms as part of a more extensive doctoral research project aimed at assessing barriers to cervical cancer screening in Adjara region and developing evidence-based recommendations to strengthen preventive services.

The target population consisted of physicians directly involved in outpatient or preventive care, namely, family physicians, gynecologists, oncologists, and internal medicine specialists, who are employed in public or private healthcare institutions across all the six municipalities noted above. The target population comprised physicians involved in outpatient/preventive care from all six municipalities of Adjara (including family physicians, gynecologists, oncologists, and internal medicine specialists). Because participation was voluntary and the exact number of invited physicians could not be determined, an exact response rate could not be calculated.

Because participation was voluntary and the exact number of invited physicians could not be determined, an exact response rate could not be calculated, and comparison with non-participants was not possible. Accordingly, the possibility of non-response bias cannot be excluded.

A focused and regionally comprehensive sampling approach was used to ensure balanced representation of urban and rural facilities. In total, 209 physicians participated voluntarily in the survey [see Supplementary Tables]. The sample size exceeded the minimum requirement (n = 180) for logistic regression with up to eight predictors and an expected response rate of 80%.

The study instrument and analytical framework were informed by the Socio-Ecological Model (SEM), which conceptualizes health behaviors as influenced by factors operating across multiple levels of the social and organizational environment [see Supplementary File S1]. Accordingly, the questionnaire was structured to capture determinants at the individual level (e.g., physicians’ attitudes toward screening and perceptions of potential harms), the interpersonal level (e.g., physician–patient communication and interaction during preventive consultations), and the organizational level (e.g., consultation time availability, collaboration patterns, and access to screening program information). This framework guided the selection and classification of explanatory variables and supported a structured interpretation of the determinants associated with screening recommendation practices. Although the statistical models estimated the independent associations between variables from these domains, the SEM primarily served as a conceptual framework for organizing determinants across levels rather than as a formal multilevel analytical model.

**Figure 1:**
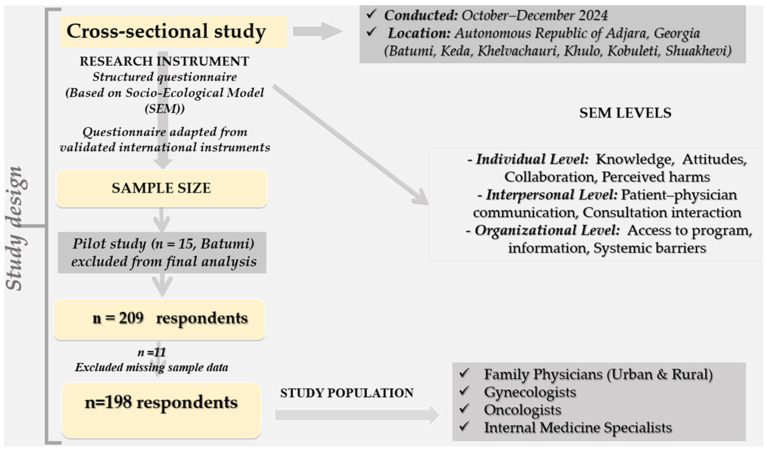
Overview of the cross-sectional study design, research instrument based on the Socio-Ecological Model (SEM), sampling procedure, and study population.

The instrument [Figure 1] was developed based on validated international studies [25,26]; however, it was adapted and maximally tailored to the Georgian healthcare context. The instrument was reviewed by the expert group of physicians and public health researchers, refined in terms of content validity, and translated into Georgian language. A pilot test was conducted with 15 physicians in the Batumi area to assess the clarity, internal consistency, and administration time; then, minor modifications were made for wording and logical flow. The pilot data were excluded from the final analysis of this study. The internal consistency of the binary barrier items was assessed using the Kuder–Richardson Formula 20 (KR-20). Additionally, because the items were designed to capture heterogeneous contextual barriers rather than a single latent construct, high internal consistency was not expected. Consequently, the KR-20 coefficient was reported descriptively, and the barrier variables were analyzed individually rather than combined into a composite scale. Given that the barrier items were developed to represent distinct contextual and organizational obstacles rather than to measure a single latent construct, internal consistency was interpreted with caution, and the barriers were analyzed primarily at the individual-item level.

The primary outcome variable was physician engagement in screening promotion, operationalized as self-reported recommendation of cervical cancer screening within the past week (coded 1 = yes, and 0 = no).

The primary outcome was self-reported recommendation of cervical cancer screening within the past week (yes/no). To provide a broader assessment of physicians’ typical screening behavior, recommendation frequency was also analyzed as a secondary ordinal outcome (always, often, sometimes, rarely, never). A secondary ordinal outcome captured recommendation frequency (“never”, “rarely”, “sometimes”, “often”, or “always”). Key explanatory variables included Gender (male or female), Specialty (gynecologist, family physician—urban, family physician—rural, internist/other, or oncologist), Years in Practice (1–3, 4–10, or ≥11 years), Practice Location (urban vs. rural, with Keda, Khulo, and Shuakhevi regions classified as rural), Adequacy of Consultation Time (sufficient vs. insufficient), and Perceived Harms of Screening (no, unsure, or yes).

### 2.2. Data Collection

The survey was conducted anonymously through medical associations, healthcare institutions, and direct public support; both printed and electronic forms were used in the research process. The survey responses were entered into a specially prepared Excel database; which was regularly cross-checked by two independent evaluators. The study protocol was approved by the Research Ethics Committee of the University of Georgia (Approval No. 007, 2024). Participation in the study was voluntary, and all responses were anonymized. The study adhered to the Declaration of Helsinki (2013 revision) and also followed the local data protection regulations.

### 2.3. Statistical Analysis

Data were analyzed using IBM SPSS Statistics v.26 (Armonk, New York, USA). The descriptive statistics (such as frequencies and percentages) summarized respondent characteristics/practice patterns. Missing data were handled using complete-case analysis (final analytic sample: n = 198). Although the proportion of missing data was small, this approach may contribute to bias if the data were not missing completely at random.

For the primary outcome—recommending screening within the past week—a multivariable logistic regression model estimated adjusted odds ratios (aOR) with 95% confidence intervals (CI). Covariates included gender, speciality, years in practice, rural vs. urban setting, adequacy of consultation time, and perceived harms of screening. Model fit was evaluated using McFadden’s pseudo-R^2^, the Akaike Information Criterion (AIC), and the Hosmer–Lemeshow goodness-of-fit test. A proportional-odds (ordinal logistic) model examined predictors of screening-recommendation frequency (1 = never → 5 = always); predicted probabilities were computed for speciality × rurality combinations. Missing values were handled through complete-case analysis (final n = 198). All tests were two-sided, with *p* < 0.05 considered statistically significant.

## 3. Results

A total of 209 physicians participated in the study, representing all six municipalities of the Adjara region with the following numbers: Batumi (59.3%), Khulo (12.9%), Shuakhevi (11%), Keda (8.1%), Khelvachauri (4.8%), and Kobuleti (3.8%). Most respondents were female (90.4%) and fell within the age range of 46 to 55 years (40.7%). The majority of respondents (79.4%) had 11 or more years of experience, while 17.2% reported 4–10 years, and 3.3% had 1–3 years of experience. Regarding the specialty, family physicians were the most common (urban 30.6%; rural 27.8%), followed by internists (20.1%), gynecologists (18.7%), and oncologists (1.9%) [Table 1].

According to our research survey, almost all participants (96.7%) reported providing cervical cancer screening to asymptomatic, and age-eligible women. The self-reported frequency of recommending screening was high, with responses of “always” (51.2%), “often” (40.2%), “sometimes” (5.7%), “rarely” (2.4%), and “never” (0.5%). Regarding ‘frequency’, a notable portion had recommended screening within the recommended intervals: 58.9% within the past week, 19.1% within the past month, 8.1% within the past three months, and 3.3% within the past six months; only 1.5% had not done so in the past year [Table 2].

The ‘perception of harms’ associated with screening was generally low, with most respondents indicating no concern (89%), while 5.7% reported problems, and 5.3% were unsure. Among those who cited harms, the most common reasons included psychological distress, physical discomfort, and financial barriers. Overall, attitudes toward primary care promotion of screening were very positive, with 91.4% of participants describing their stance as positive or very positive. Additionally, agreement with the preventive health statement was high, with 88% agreeing and 8.6% partially agreeing [Table 3].

Physicians specified several patient-level and organizational barriers. The most common barriers were lack of patient information (37.3%), stigma or fear (26.3%), and geographic inaccessibility (16.3%). Less frequent barriers included lack of time (9.1%) and low patient trust (7.2%). At the organizational level, 14.8% of survey participants reported difficulty accessing program information, and 21.1% experienced partial problems, mainly due to delayed updates (10.5%), inaccurate information (10%), and administrative inefficiencies (9.6%) [Table 4].

Inter-professional collaboration was reported as frequent by 59.3% of respondents and occasional by 19.6%. This collaboration mainly occurred through patient referrals (64.6%), data sharing (10%), and joint meetings (7.2%). The primary sources of information about screening programs were medical conferences or literature (45.5%) and healthcare administration (30.1%). Physicians most commonly recommended the following priorities for improvement: public awareness campaigns (29.7%), enhanced service accessibility (26.3%), provider training (21.1%), and improved program management (17.7%) [Table 4]. The internal consistency of the binary barriers index was moderate, with a Krippendorff’s alpha (KR-20) of 0.48. This supports the idea that the items in question reflect different contextual challenges rather than a single underlying construct.

A multivariable logistic regression analysis was performed to identify predictors linked to the recommendation of cervical cancer screening within the past week (complete-case N = 198) [Table 5]. The results show that uncertainty about the harms related to screening was independently associated with lower odds of giving a recent recommendation compared to those who perceived no harms (aOR = 0.42, 95% CI 0.21–0.88, *p* = 0.023).

In comparison to gynecologists, family physicians—both in rural (aOR = 0.51, 95% CI = 0.28–0.93, *p* = 0.029) and urban settings (aOR = 0.58, 95% CI = 0.33–0.99, *p* = 0.047)—demonstrated a significantly lower likelihood of recommending screening. Furthermore, a positive correlation was observed between adequate consultation time and recent screening recommendations (aOR = 1.67, 95% CI = 0.98–2.85, *p* = 0.058). Rural practice, independent of speciality, was associated with decreased engagement with screening recommendations (aOR = 0.60, 95% CI = 0.34–1.09, *p* = 0.095), while clinician gender and years of practice did not reach statistical significance. The overall model demonstrated acceptable fit, as indicated by McFadden pseudo-R^2^ = 0.14; AIC = 188.4; and Hosmer–Lemeshow test *p* = 0.61. However, the relatively low pseudo-R^2^ value indicates that a substantial proportion of the variation in physicians’ screening recommendation behavior remains unexplained.

According to our study, ordinal logistic regression with recommendation frequency (as the outcome) produced results consistent with the primary model. Uncertainty about screening harms and rural practice is inversely related to recommendation intensity, while adequate consultation time increased the likelihood of “always” recommending screening. Predicted probabilities indicated that rural family physicians had the lowest likelihood of consistently recommending screening (*p* = 0.47) compared with gynecologists (*p* = 0.83) and urban family physicians (*p* = 0.61). No violation of the proportional-odds assumption was detected (Brant test *p* = 0.72).

Based on our research analysis, almost all doctors confirmed their support and facilitation of cervical cancer screening. However, uncertainty about the harms of screening and rural practices significantly limits the participation in cervical cancer screening. Sufficient time for consultation has become a positive, almost determinative factor in the conclusion of preventive counseling. Based on the above, the importance (necessity) of informational clarity and of resolving structural barriers is confirmed to increase doctors’ participation in promoting cervical cancer screening in Adjara and to achieve significant results.

## 4. Discussion

This study examined physician-level and organizational factors associated with cervical cancer screening recommendation practices in the Adjara region of Georgia. Although physicians generally reported positive attitudes toward screening, variation in the frequency of recommendations was associated with factors such as consultation time, perceived uncertainty about screening harms, and practice setting. These findings suggest that preventive counseling practices may be related not only to physicians’ attitudes but also to broader organizational and informational conditions within the clinical settings. These findings highlight perceived barriers and contextual factors that may influence screening recommendation practices and warrant further investigation in future research. This study demonstrates that the majority of physicians in the Adjara region of Georgia have a positive attitude towards cervical cancer screening and recognize its importance in preventive health care. Nevertheless, the practice of recommending screening is not consistent and varies significantly by specialty and practice location. In particular, family physicians, especially those working in rural areas, were less likely than gynecologists to recommend screening. In addition, uncertainty about the potential harms associated with screening and insufficient time for consultation were independently associated with reduced frequency of screening recommendations. Despite the significant benefits of screening, inequalities in access persist due to multiple factors (cultural, socio-economic, and other systemic). Focusing on improving the current state of cervical cancer screening practices and reducing existing barriers will significantly impact outcomes in a positive context [27,28].

It is worth noting that our survey identified a lack of information (37.3%), stigma or fear (26.3%), and problems accessing services due to location (16.3%). Among the less important problems are limited doctors’ time (9.1%) and patient distrust (7.2%). Among the organizational challenges, it is worth noting the difficulty faced by staff in accessing program information (14.8%), as well as partial problems such as delays in updating (10.5%), incorrect data (10%), and inefficient administration (9.6%).

The vast majority of participants always offer cervical cancer screening services to asymptomatic women (who meet the relevant criteria). This high level of attention confirms the respondents’ attitude towards preventive health practices. In addition, more than half of the participants (51.2%) stated that they “always” recommend screening, and an additional 40.2% do so “often”. Only a small minority reported rarely or never recommending it. It is also noteworthy that the recommendations’ timing closely aligns with established screening guidelines. Overall, our data indicates that healthcare providers support cervical cancer screening, which will play an essential role in earlier detection and enhanced treatment outcomes for patients.

According to a study by Hu and colleagues [27], among the rural Chinese population, primary health care physicians show a significant intention to provide cervical cancer screening services; attitude and related knowledge significantly and positively affect this intention. However, low levels of perceived behavioral control indicate a lack of time/equipment/training; overall, the attitude of primary health care physicians to provide cervical cancer screening services to rural women is positive [29].

Moreover, the concerns about harms associated with screening are minimal according to respondents involved in the survey; moreover, the absolute majority of problems reported the absence of a problem; while those who expressed concerns were mainly related to psychological distress, physical discomfort and financial barriers. It is noteworthy that in the case of the Adjara population, the attitude towards the promotion of screening in primary health care is positive and there is a strong agreement with preventive health; accordingly, it is confirmed that there is noticeable support for screening initiatives.

A study of barriers and attitudes towards cervical cancer screening, specifically sociodemographic factors, screening behavior, and awareness (384 female respondents aged 25–64 years from primary health care facilities in São Paulo, Brazil), where participants in the study assessed barriers and attitudes using a five-point Likert scale (which was later summarized on a three-point scale for analysis). The main personal and structural barriers included fear (41%), delayed test results (30%), long waiting times (30%), and shame (29%). Notably, a significant association was found between low income and delayed test results/long waiting times, as well as between low education and the experience of delays and shame. In addition, overall positive attitudes towards screening exceeded 95%, especially among women who understood the role of the smear in early detection and the possibility of survival; despite the high participation rate in screening, persistent socio-economic inequalities in the access and personal barriers clearly highlighting the need for interventions. Accordingly, strengthening the primary health care system with maximum patient-centered approaches will promote screening adherence and reduce existing inequalities [30].

According to our research, collaboration between respondents (depending on the profession) is relatively frequent, mainly due to patient referrals/data sharing/joint meetings. Information about screening programs is mainly shared through medical conferences, literature, and health administration sources. Physicians prioritize public awareness campaigns/service accessibility/provider training and improving program management associated with screening. Furthermore, the multifaceted nature of the barriers is also noteworthy.

The demographic profile of the physician participants suggests that the healthcare workforce in Adjara is predominantly composed of experienced female practitioners. Our findings emphasize the pivotal role of family medicine in both urban and rural settings, with a strong emphasis on primary care. Also, uncertainty regarding screening harms and rural practices was inversely related to the intensity of the recommendation, whereas adequate consultation time increased the likelihood of screening being “always” recommended.

In general, clinics with established cancer prevention programs have adequate resources available, and therefore more physicians are actively involved in screening [31]. Notably, the health system approach generally enhances collaboration among health professionals; therefore, it is more likely to improve screening practices by reducing professional workload and improving approaches and relationships with patients [32]. In turn, existing socio-cultural norms and societal perceptions may indirectly influence physicians through patient attitudes and behaviors. Particularly, where health-related misconceptions are prevalent (including stigma surrounding cancer screening), physicians may find it significantly more difficult to promote the practice. Accordingly, more educational programs about cervical cancer screening are needed, and maximum public involvement can significantly reduce existing barriers or misconceptions [33,34].

Physicians play an essential role in cervical cancer screening; in particular, their training, attitude, and well-focused recommendations significantly influence the success of the screening process; therefore, within the framework of the socioecological model, it is clear that patient characteristics and clinic infrastructure influence aspects related to the provider. Of course, health policy is a priority in this issue, the role of which is fundamental. By coordinating screening/issuing recommendations, more opportunities are created to improve screening outcomes [35].

Our study found that both rural and urban family physicians were significantly less likely to recommend screening than gynecologists. In addition, there was a positive correlation between adequate consultation time and recent screening recommendations. Our study results show that the vast majority of physicians are supportive of cervical cancer screening; however, potential side effects associated with screening, as well as challenges in rural settings, significantly hinder the process of screening. It is also noteworthy that providing sufficient consultation time is a crucial aspect of the success of preventive counseling. Based on the above, it is clear that ensuring the accuracy of information and addressing structural barriers will maximize physicians’ participation in cervical cancer screening advocacy in Adjara, ultimately achieving long-term screening effectiveness.

Our findings suggest that differences in screening recommendation practices may be associated with the structural and organizational conditions within the routine clinical care (including consultation time and the clarity of screening guidelines, rather than with physicians’ attitudes alone). These results highlight the importance of considering organizational aspects when examining factors related to the promotion/enhancement of cervical cancer screening recommendations.

Although there were differences in screening recommendation practices across physician specialties, the overall level of recommendation was high (in particular, more than 90% of physicians indicated that they “often” or “always” recommend cervical cancer screening to patients when appropriate). Therefore, greater caution is warranted regarding the specialty-based differences. The lower likelihood of recommendation among family physicians compared with gynecologists may therefore be related to differences in clinical roles and approaches to patient care, rather than to differences in physicians’ commitment to promoting/supporting screening. Gynecologists may be more likely to encounter patients in reproductive health settings where cervical cancer screening is discussed more routinely. Family physicians may address a broader range of health concerns within the limited consultation time.

The results suggest that insufficient screening coverage cannot be attributed just to doctors’ lack of knowledge/motivation. Despite strong support, the successful implementation of established preventative recommendations is usually limited by day-to-day clinical and organizational challenges. Similarly to our results, the gap between attitudes and actual practice has been described in other countries; in this context, it is particularly noteworthy in primary care settings, where workload is noticeably high and time is limited. These circumstances ultimately limit the opportunities for suitable preventive consultation [29].

One of the main findings of the investigation is the impact of uncertainty about possible harms associated with screening on physicians’ recommendation behavior. It is notable that among physicians who reported uncertainty about potential harms, screening recommendations were less frequent. In contrast, the perception of harm itself was not associated with a decrease in recommendations. Therefore, the delay in preventive practice may be more closely connected to inaccurate information or an ambiguous perception of clinical recommendations than to direct resistance to screening. According to other investigations, underscoring the importance of physicians clearly communicating risks and benefits [36].

We recommend that the linkage between uncertainty about screening harms and low rates of screening recommendations should be approached with caution. Regardless, uncertainty may also reflect gaps in understanding or guidelines; also, alternative explanations include defensive clinical practice, ambiguity in screening recommendations, or variability in communication from health authorities. Furthermore, the proportion of physicians reporting uncertainty was relatively small. Therefore, future research should be designed to minimize these shortcomings.

Physician “harm uncertainty” (also associated with a lower likelihood of recommending cervical cancer screening) may partly reflect the rural-urban differences in recommendation behavior. In addition to difficulties with guidelines or follow-up mechanisms, reduced clinician confidence may lead to more conservative decisions, especially when physicians anticipate difficulties with follow-up after a positive result. Thus, one possible reason for the high level of uncertainty in rural settings is limited access to continuing medical education (CME)/professional information exchange. In addition, uncertainty may also reflect system-level limitations (including follow-up mechanisms, limited capacity to manage positive screening results, etc.). It is clear that educational as well as structural factors are the main drivers of preventive practices.

The differences determined by specialty and practice location emphasize the role of structural factors in encouraging screening. In particular, family physicians, particularly those working in rural settings, were less likely to recommend screening, which high patient volumes, limited referral capability, and lower access to screening services may explain. Similar regional differences have been described in other countries and reflect systemic difficulties specific to rural practices [26].

According to the study, sufficient consultation time was identified as an important contributing factor to screening recommendations. In particular, physicians who allocated adequate time to patient interaction were more likely to recommend screening. In addition to the above, time constraints have been identified as an essential barrier to preventive consultation in primary care settings and in the settings where screening programs are formally available [37]. The use of a socio-ecological model enabled us to identify barriers to screening uptake; screening constraints are heterogeneous and include individual/organizational/multiple systemic factors. Overall, the it is suggesting that screening is conducted under unequal conditions [38].

The present study has several limitations—screening recommendation practices were assessed using self-reported data; accordingly, they may be subject to social desirability bias and could overestimate actual clinical behavior. The study was conducted in a single region of Georgia (viz., Adjara), which may limit generalizability to the national level. In addition, some contextual barriers were measured using single-item indicators rather than validated multi-item scales. Thus, future studies must include objective measures and include broader geographic samples.

In our study, certain limitations should be considered. Social factors may influence screening recommendation behavior. Also, the cross-sectional study design does not allow for the determination of cause-and-effect relationships. It should be noted that the methodological limitation is linked to the one-week recall period used to evaluate screening recommendations. Although this measure reflects recent clinical activity, it may be influenced by short-term workload or patient-mix fluctuations and may not fully represent routine practice. Therefore, an ordinal measure of recommendation frequency was included to provide a broader assessment of physicians’ typical screening behavior. The KR-20 coefficient for the barrier items was 0.48. Although this value falls below the conventional thresholds for psychometric scales, it likely reflects the conceptual heterogeneity of the items, which represent multiple independent contextual barriers to screening implementation. Accordingly, the items were interpreted as separate indicators rather than as elements of a single internally consistent scale. The binary barriers index showed low internal consistency (KR-20 = 0.48), which likely reflects the heterogeneous nature of the barriers assessed. These items were designed to identify various contextual barriers rather than assess a singular latent construct. Therefore, they should be regarded as distinct indicators of barriers rather than as components of a cohesive scale.

It is noteworthy that the model showed acceptable fit (McFadden pseudo-R^2^ = 0.14; AIC = 188.4; Hosmer–Lemeshow *p* = 0.61). At the same time, the relatively low pseudo-R^2^ indicates that a substantial proportion of the variation in physicians’ screening recommendation behavior remains unexplained.

Additional contextual factors, such as clinic type, patient volume, organizational workload, and others, may influence physicians’ screening recommendation practices and may act as potential confounders. These factors were not captured in the present survey and should be considered in future research.

It should be noted that the relatively lower likelihood of screening recommendation among family physicians may partially mediate structural characteristics of primary care practice (including broader clinical responsibilities and time constraints), which compared with specialty care settings, may define possibilities for preventive counseling.

We suggest that future studies with larger samples could explore potential interaction effects among contextual variables, and could also apply multilevel modelling to capture hierarchical structures within healthcare systems better. Thus, additional contextual factors (such as clinic workload, availability of screening services, referral pathways, local program support, and others) may also substantially influence physicians’ screening recommendation practices. These factors were not captured in the present survey and should be explicitly considered in future research investigations.

It should be noted that the relatively lower likelihood of screening recommendations among family physicians may partly reflect structural characteristics of primary care practice, including broader clinical responsibilities, time constraints, and differences in access to screening-related resources and professional training.

As mentioned, the screening practices were self-reported and may therefore be subject to social desirability bias. Overall, the cross-sectional design precludes causal inference; therefore, caution should be exercised in determining the facts. The one-week recall window may capture short-term fluctuations rather than routine practices. In addition, the small, specialized subgroups limit statistical power, and the study was conducted at the level of a single region, limiting generalizability.

The study relied on self-reported physician behavior, which may be affected by social desirability bias and may not fully reflect actual clinical practice. It should be noted that future research may strengthen validity through triangulation (including by comparing survey responses with objective data sources such as clinical records or patient-reported data).

Thus, the relatively low internal consistency of the barrier subset likely reflects the heterogeneous nature of the included barriers. Future studies with larger sample sizes and more comprehensive methodological approaches may refine the instrument further, including through the development of separate subscales or the application of factor analysis.

## 5. Conclusions

Thus, this study examined physician-level and organizational factors associated with cervical cancer screening recommendation practices in the Adjara region of Georgia. Although physicians generally reported positive attitudes toward screening, variation in the frequency of recommendations was associated with such factors as consultation time, perceived uncertainty about screening harms, and practice setting. These findings suggest that preventive counseling practices may be related not only to physicians’ attitudes but also to broader organizational and informational conditions within clinical settings. These findings also highlight perceived barriers and contextual factors that may influence screening recommendation practices and warrant further research investigation.

## Figures and Tables

**Table 1:** Demographic and professional characteristics of participating physicians.

Variable	Category	n	%
Municipality	Batumi	124	59.3
	Khulo	27	12.9
	Shuakhevi	23	11.0
	Keda	17	8.1
	Khelvachauri	10	4.8
	Kobuleti	8	3.8
Gender	Female	189	90.4
	Male	20	9.6
Years in Practice	1–3	7	3.3
	4–10	36	17.2
	≥11	166	79.4
Speciality	Family physician (urban)	64	30.6
	Family physician (rural)	58	27.8
	Internist	42	20.1
	Gynecologist	39	18.7
	Oncologist	4	1.9

**Table 2:** Cervical cancer screening recommendation for frequency and timing.

Measure	Response	n	%
Offer to asymptomatic women	Yes	202	96.7
	No	7	3.3
Recommendation frequency	Always	107	51.2
	Often	84	40.2
	Sometimes	12	5.7
	Rarely	5	2.4
	Never	1	0.5
Last recommendation	Past week	123	58.9
	Past month	40	19.1
	3 months	17	8.1
	6 months	7	3.3
	1 year	1	0.5
	≥2 years	2	1.0
	Other	19	9.1

**Table 3:** Perceptions, attitudes, and agreement with preventive statements.

Measure	Category	n	%
Perceived harms	No	186	89.0
	Yes	12	5.7
	Unsure	11	5.3
Attitude to primary health care promotion	Very positive	90	43.1
	Positive	101	48.3
	Neutral	18	8.6
	Negative	0	0
Agreement with the preventive statement	Agree	184	88.0
	Partly agree	18	8.6
	Disagree	7	3.3

**Table 4:** Barriers, collaboration patterns, and improvement priorities.

Domain	Item	n	%
Patient barriers	Lack of patient information	78	37.3
	Stigma/fear	55	26.3
	Geographic inaccessibility	34	16.3
	Lack of time	19	9.1
	Lack of trust in screening	15	7.2
Organizational barriers	Lack of timely updates	22	10.5
	Insufficient/inaccurate information	21	10.0
	Administrative delays	20	9.6
Collaboration forms	Patient referral	135	64.6
	Data sharing	21	10.0
	Joint meetings	15	7.2
Improvement priorities	Awareness campaigns	62	29.7
	Improving access	55	26.3
	Provider training	44	21.1
	Program management/monitoring	37	17.7

**Table 5:** Summary of multivariable logistic regression analyses of the data: adjusted odds ratios (aOR), 95% confidence interval (CI), and *p*-values for key predictors.

Predictor	aOR	95% CI	*p*-Value
Female vs. male	1.02	0.54–1.91	0.94
Family physician (urban) vs. Gynecologist	0.58	0.33–0.99	**0.047**
Family physician (rural) vs. Gynecologist	0.51	0.28–0.93	**0.029**
Internist/other vs. Gynecologist	0.74	0.41–1.35	0.33
Oncologist vs. Gynecologist	1.22	0.55–2.77	0.63
Years ≥ 11 vs. 1–3	1.31	0.56–3.08	0.53
Adequate consultation time (Yes vs. No)	1.67	0.98–2.85	0.058
Rural practice (vs. Urban)	0.60	0.34–1.09	0.095
Perceived harms: Unsure vs. No	0.42	0.21–0.88	**0.023**
Perceived harms: Yes vs. No	1.42	0.56–3.64	0.46

Model fit: McFadden pseudo-R^2^ = 0.14; AIC = 188.4; Hosmer–Lemeshow *p* = 0.61. *p*-Values on bold-font reflect statisically significant.

## Data Availability

The data presented in this study are available on reasonable request from the corresponding authors.

## Supplementary Materials

The following supporting information can be downloaded at https://www.mdpi.com/article/10.3390/healthcare14070961/s1. File S1: Physician Questionnaire; Table S1: Physicians’ responses regarding consultation duration sufficiency and cancer screening recommendations, stratified by gender; Table S2: Frequency of patient inquiries regarding screening and the recommendations provided by physicians, categorized by gender; Table S3: Comparison of Time Since the Last Physician-Recommended Cancer Screening by Gender; Table S4: Gender Differences in Access to Screening Information and Collaborative Practices Among Healthcare Professionals in Cancer Screening Programs; Table S5: Gender Differences in Attitudes Toward Cancer Screening and Preferred Screening Priorities in Primary Health Care Settings; Table S6: Gender Differences in Sources of Information on Cancer Screening Programs Among Physicians.
