# Supplementary material for: Physician-Level Determinants of Cervical Cancer Screening Practices: A Socio-Ecological Model-Based Study from Adjara, Georgia
Source: Healthcare (Basel). 2026 Apr 6;14(7):961. doi: 10.3390/healthcare14070961 (PMC13073022; doi:10.3390/healthcare14070961)
Supplement: Supplementary file 1 [file healthcare-14-00961-s001.zip › Supliment 1.pdf]

Supplement 1. Physician Questionnaire

(Social Ecological Model – SEM–based instrument used to assess physicians' attitudes, practices, and perceived barriers toward cervical cancer screening in Adjara, Georgia)

Section 1. Demographic and Professional Information

1. **Your primary place of work (municipality):**
   ☐ Batumi
   ☐ Kobuleti
   ☐ Khelvachauri
   ☐ Keda
   ☐ Shuakhevi
   ☐ Khulo
2. **Your specialty:**
   ☐ Family physician (urban practice)
   ☐ Family physician (rural ambulatory)
   ☐ Gynecologist
   ☐ Oncologist
   ☐ Internist (endocrinologist, gastroenterologist, or other)
3. **Years of professional experience:**
   ☐ 1–3 years
   ☐ 4–10 years
   ☐ ≥11 years
4. **Your age:**
   ☐ 25–35
   ☐ 36–45
   ☐ 46–55
   ☐ 56–65
5. **Gender:**
   ☐ Male
   ☐ Female

Section 2. Clinical Practice and Screening Promotion

6. **In your opinion, is the duration of a standard consultation sufficient to discuss the importance of cancer screening with a patient?**
   ☐ Yes
   ☐ No

7. **Do you offer cancer screening to asymptomatic patients who fall within the recommended age or risk groups?**
   ☐ Yes
   ☐ No

8. **If "No," please specify the main reason(s):**

---

9. **How often do patients ask you about the need for cancer screening?**
   ☐ Always
   ☐ Often
   ☐ Sometimes
   ☐ Rarely
   ☐ Never

10. **How often do you recommend participation in cancer screening to your patients?**
    ☐ Always
    ☐ Often
    ☐ Sometimes
    ☐ Rarely
    ☐ Never

11. **When was the last time you advised a patient to undergo screening?**
    ☐ Within the past week
    ☐ Within the past month
    ☐ Within the past 3 months
    ☐ Within the past 6 months
    ☐ Within the past year
    ☐ More than 2 years ago
    ☐ Other (please specify): ______________________________

12. **Please describe a specific case where a patient's screening participation affected the outcome:**
    ☐ Early-stage cancer detected and successfully treated
    ☐ Delayed diagnosis due to lack of screening
    ☐ Patient participation led to improved health outcomes
    ☐ Other (please specify): ______________________________

Section 3. Perceptions, Attitudes, and Beliefs

13. **Do you believe that cancer screening procedures can be harmful to patients?**

☐ Yes
☐ No
☐ Not sure

14. **If "Yes," please indicate the type of potential harm:**
☐ Physical harm
☐ Psychological distress
☐ Financial burden
☐ Other (please specify): ______________________________

15. **What impact have national cancer screening programs had on the Georgian population?**
☐ Positive
☐ Negative
☐ No impact

16. **What do you think should be improved or changed in cancer screening programs?**
☐ Public awareness and information campaigns
☐ Accessibility of screening services (geographical or financial)
☐ Training of medical personnel
☐ Increased financial incentives for physicians
☐ Improvement of diagnostic and technical equipment
☐ Better management and monitoring of screening programs
☐ Other (please specify): ______________________________

17. **What is your primary source of information about screening programs?**
☐ Health administration
☐ Colleagues
☐ Social media
☐ Medical conferences or professional literature
☐ Other (please specify): ______________________________

18. **Do you experience difficulties in obtaining information about screening programs?**
☐ Yes
☐ No
☐ Partly

19. **If "Yes" or "Partly," please specify the main issue:**
☐ Limited access to information (geographical or technical)
☐ Inaccurate or insufficient information
☐ Lack of timely updates

☐ Inefficient communication between administration and physicians
☐ Other (please specify): ______________________________

Section 4. Collaboration and Professional Environment

20. **Do you collaborate with other healthcare professionals involved in cancer screening programs?**
☐ Yes
☐ No
☐ Sometimes

21. **If "Yes" or "Sometimes," indicate the type of collaboration:**
☐ Patient referral
☐ Data sharing or reporting
☐ Joint meetings or discussions
☐ Other (please specify): ______________________________

22. **How would you describe your personal attitude toward the promotion of cancer screening by primary healthcare?**
☐ Very positive
☐ Positive
☐ Neutral
☐ Negative
☐ Very negative

23. **Do you agree with the following statement:**
"Preventive measures are implemented to avoid disease or to reduce the risk of its consequences (e.g., vaccination, balanced work and rest schedule, healthy diet, physical activity, and healthy environment)."
☐ Yes
☐ No
☐ Partly

Section 5. Barriers and Patient-Related Observations

24. **In your opinion, what are the main barriers patients face when participating in cancer screening programs?**
☐ Geographical inaccessibility of services
☐ Lack of information
☐ Stigma or fear
☐ Lack of trust in screening

☐ Lack of time
☐ Other (please specify): ______________________________

25. **Which screening program has the highest participation among your patients?**
☐ Cervical cancer
☐ Breast cancer
☐ Colorectal cancer
☐ Not sure
