# Supplementary material for: Physician-Level Determinants of Cervical Cancer Screening Practices: A Socio-Ecological Model-Based Study from Adjara, Georgia
Source: Healthcare (Basel). 2026 Apr 6;14(7):961. doi: 10.3390/healthcare14070961 (PMC13073022; doi:10.3390/healthcare14070961)
Supplement: Supplementary file 1 [file healthcare-14-00961-s001.zip › Suplimentary tables.pdf]

Supplementary Tables

No. 1. Physicians' responses regarding consultation duration sufficiency and cancer screening recommendations, stratified by gender

In your opinion, is the duration of the consultation sufficient to talk to the patient about the importance of screening?			
**Gender**	**Yes**	**No**	**Total**
Male	80.0%	20.0%	100.0%
Famle	77.8%	22.2%	100.0%
Do you offer asymptomatic patients who fall into the recommended age or risk groups to participate in a cancer screening program?			
**Gender**	**Yes**	**No**	**Total**
Male	96.8%	3.2%	100.0%
Famle	96.7%	33%	1000%

No.2. Frequency of patient inquiries regarding screening and the recommendations provided by physicians, categorized by gender.

How often do patients ask you about the need for screening?						
**Gender**	**Never**	**Sometimes**	**Rarely**	**Always**	**Often**	
Male	5.0%	15.0%	40.0%	15.0%	25.0%	1000%
Famale	0	27.0%	31.2%	12.2%	29.6%	100.0%
How often do you recommend patients participate in cancer screening?						
**Gender**	**Never**	**Sometimes**	**Rarely**	**Always**	**Often**	
Male	5.0%	20.0%	0	60.0%	15.0%	100.0%
Famale	0	4.2%	2.6%	50.3%	42.9%	100.0%

No. 3 Comparison of Time Since the Last Physician-Recommended Cancer Screening by Gende

Please specify when you last advised the patient to undergo screening.								
**Gender**	**2 Years ago**	**3 years ago**	**6 Years ago**	**One month ago**	**One week ago**	**One years ago**	**Others**	**Total**
Male	0	15.0%	0	25.0%	60.0%	0	0	100%
Famale	1.1%	7.4%	3.7%	18.5%	58.7%	5%	10.1%	100%

No 4. Gender Differences in Access to Screening Information and Collaborative Practices Among Healthcare Professionals in Cancer Screening Programs

Do you have trouble finding information about screening programs?				
**Gender**	**No**	**Yes**	**Partly**	
Male	40%	35%	25%	100%
Female	50.8%	28.6%	20.6%	100%

Do you collaborate with other healthcare professionals involved in the cancer screening program?				
**Gender**	**No**	**Yes**	**Sometimes**	
Male	25%	60%	15%	100%
Female	12.7%	67.2%	20.1%	100%

No. 5 Gender Differences in Attitudes Toward Cancer Screening and Preferred Screening Priorities in Primary Health Care Settings

Describe your personal attitude towards promoting cancer screening programs through primary health care?						
**Gender**	**Very negative**	**Negative**	**Neutral**	**Positive**	**Very positive**	**Total**
Male	5%	0%	15%	25%	55%	100%
Female	0%	0.5%	6.9%	50.8%	41.8%	100%

No. 6. Gender Differences in Sources of Information on Cancer Screening Programs

What do you think is the source of information for doctors about screening programs?						
**Gender**	**Administration**	**Colleagues**	**Conference or medical literature**	**Social media**	**Others**	**Total**
**Male**	45%	10%	25%	20%	0 %	100%
**Female**	28.6%	7.9%	47.6%	10.1%	5.8%	100%

Among Physicians

Describe your personal attitude towards promoting cancer screening programs through primary health care?						
**Gender**	**I have no information**	**Cervical cancer**	**Breast cancer**	**Colorectal cancer**	**Other**	**Total**
**Male**	15%	45%	25%	10%	5%	100%
**Female**	14.3%	34.9%	46.6%	2.6%	1.6%	100%
